# Biogeography of the West Indies: A complex scenario for species radiations in terrestrial and aquatic habitats

**DOI:** 10.1002/ece3.7236

**Published:** 2021-02-10

**Authors:** Rodet Rodriguez‐Silva, Ingo Schlupp

**Affiliations:** ^1^ Department of Biology University of Oklahoma Norman OK USA

**Keywords:** biogeography, island, livebearing fishes, West Indies

## Abstract

Studies of the biogeography of the West Indies are numerous but not all taxonomic groups have received the same attention. Many of the contributions to this field have historically focused on terrestrial vertebrates from a perspective closely linked to the classical theory of island biogeography. However, some recent works have questioned whether some of the assumptions of this theory are too simplistic. In this review, we compiled information about the West Indies biogeography based on an extensive and rigorous literature search. While we offer some background of the main hypotheses that explain the origin of the Caribbean biota, our main purpose here is to highlight divergent diversification patterns observed in terrestrial versus aquatic groups of the West Indian biota and also to shed light on the unbalanced number of studies covering the biogeography of these groups of organisms. We use an objective method to compile existing information in the field and produce a rigorous literature review. Our results show that most of the relevant literature in the field is related to the study of terrestrial organisms (mainly vertebrates) and only a small portion covers aquatic groups. Specifically, livebearing fishes show interesting deviations from the species‐area relationship predicted by classical island biogeography theory. We found that species richness on the Greater Antilles is positively correlated with island size but also with the presence of elevations showing that not only island area but also mountainous relief may be an important factor determining the number of freshwater species in the Greater Antilles. Our findings shed light on mechanisms that may differently drive speciation in aquatic versus terrestrial environments suggesting that ecological opportunity could outweigh the importance of island size in speciation. Investigations into freshwater fishes of the West Indies offer a promising avenue for understanding origins and subsequent diversification of the Caribbean biota.

## Graphical abstract

Studies about the biogeography of the West Indies are numerous but not all taxonomic groups have received the same attention. In this review, we analyze and discuss the main hypotheses for the origin of the Caribbean biota and examine both classical and less studied examples of species radiations in the West Indies. We also use an objective method to compile existing information in the field and produce a rigorous literature review about the biogeography of the West Indies.

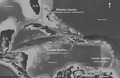

## 1 INTRODUCTION

The Caribbean region stands out as a leading biodiversity hotspot in the world exhibiting high levels of diversity and endemism both in flora and fauna (Myers et al., 2000). Particularly in the islands of the West Indies, which include the archipelagoes of the Bahamas, the Greater Antilles, and the Lesser Antilles (Berman, 2008; Hofmann, 2008), the presence of a very distinctive biota has resulted from a unique and complex combination of geological events and tropical climatic conditions. These two elements have been shown to be a major cause of diversification and speciation in reptiles (Gifford & Larson, 2008; Glor et al., 2004), amphibians (Alonso et al., 2012; Rodríguez et al., 2010), and freshwater fishes (Doadrio et al., 2009; Ponce de León et al., 2014; Rivas, 1958; Rosen & Bailey, 1963). Furthermore, it is responsible for an extraordinary diversity and endemism in several genera of plants (Santiago‐Valentin & Olmstead, 2004).

The West Indies represent a geographically complex region that provides a suitable setting to study the colonization and diversification processes that resulted in a plethora of animal and plant species currently occurring in the insular Caribbean. Although several taxa exhibit radiations mainly in the Greater Antilles, the speciation process has not been homogeneous for all groups in the Caribbean. Even though the majority of terrestrial vertebrates show higher diversification in larger islands (Hedges, 1996a; Losos, 1996, 2009), thereby confirming the positive species‐area relationship predicted by the theory of island biogeography (MacArthur & Wilson, 1967), some deviations from theory are observed in aquatic groups such as some genera of livebearing fishes (Poeciliidae). According to Ricklefs and Bermingham (2008), the West Indies are ideal natural laboratories for biogeographic and evolutionary analyses since a series of combined factors such as distance to sources of colonists as well as age and different sizes of the islands allow us to explore the evolution of several groups under diverse scenarios.

Indeed, one of the most dynamic debates in biogeography is related to the different hypotheses of colonization of the West Indies, and particularly the Greater and Lesser Antilles. It has been a contentious topic for more than a century with hypotheses that emphasize the important role of land connections and vicariance (Barbour & Matthew, 1916; Rosen, 1975; Schuchert, 1935) to studies suggesting that transoceanic dispersal has been a significant component in the origin and establishment of the insular Caribbean biota (Darlington, 1957; Hedges et al., 1992; Simpson, 1956; Williams, 1989) (Table 1). Certainly, the study of species composition and phylogenetic relationships in extant terrestrial and freshwater organisms of the West Indies can shed light on the history of the extant Caribbean biota. One reason for this is because these species depend on passive dispersal due to their limited ability to cross transoceanic barriers.

**TABLE 1:** Main biogeographic models that explain the origin of the biota of the West Indies

Biogeographic Model	Explanation	Study	Supporting evidence
Vicariance	Mechanism based on the plate‐tectonics theory. It states the presence of a direct land connection between the Proto‐Antilles and continental North and South America	Rosen (1975) Page and Lydeard (1994) Crother and Guyer (1996)	Freshwater fishes (i.e. *Ophisternon*, *Gambusia*). Also some genera of amphibians and reptiles which argues Hedges et al.’s (1992) study (i.e., *Eleutherodactylus, Anolis*, *Spherodactylus*)
Overwater dispersal	Transoceanic dispersal following the Caribbean current, which has been observed by means of natural rafts. The current moves northwestward through the Caribbean Sea from the equatorial Atlantic Ocean via the North Equatorial, North Brazil, and Guiana currents	Schuchert (1935) Hedges et al. (1992) Hedges (1996c) de Queiroz (2005) Heinicke et al. (2007) Palacios et al. (2016) Reznick et al. (2017)	Several groups of Caribbean vertebrates (i.e. small mammals, *Anolis* lizards, *Eleutherodactylus* frogs, *Limia* and *Gambusia* fishes
GAARlandia	Vicariance hypothesis that explains the colonization of the Caribbean by means of a land bridge connection that supposedly connected the Greater Antilles with South America	Iturralde‐Vinent and MacPhee (1999) Heinicke et al. (2009) Crews and Gillespie (2010) Rican et al. (2013) Matos‐Maraví et al. (2014) Weaver et al. (2016)	Tested in individual lineages of different groups (i.e. freshwater fishes of Poeciliidae and Cichlidae, butterflies of Nymphalidae, spiders of Araneae) *Eleutherodactylus* frogs

Note

Some lines of evidence and studies that support each model are also included.

Even though this review provides some context of both geological and biogeographic elements that may explain the origin and diversification of noticeable groups that have radiated mostly in the Greater Antilles, this paper does not intend to accomplish an exhaustive analysis about the biogeography of the West Indies. Actually, there are numerous works including research articles, book chapters, and entire books that examine in detail several aspects of the biogeography of the West Indies including the paleogeography of this complex geographic region, causes of diversification of several groups of organisms and also conservation problems of the biodiversity in the Caribbean islands (Crews & Esposito, 2020; Crother & Guyer, 1996; Hedges, 2001; Iturralde‐Vinent & MacPhee, 1999; Ricklefs & Bermingham, 2008; Santiago‐Valentin & Olmstead, 2004). Instead, our review paper aims to highlight contrasting biogeographical trends seen in different taxonomic groups distributed in the West Indies (specifically in the Greater Antilles), and also shed light on the unbalanced number of studies covering the biogeography of terrestrial versus aquatic organisms. For this, we use an objective method to gather existing information in the field and produce a rigorous literature review (Haddaway et al., 2020). This paper is particularly relevant in highlighting divergent diversification patterns that might occur in terrestrial versus aquatic groups of the West Indies biota. Here, we analyze both classic and scarcely studied examples of species radiations in the West Indies. Finally, this study sheds light on the importance of this region for the conservation of biodiversity.

## 2 METHODS FOR LITERATURE SEARCH

**FIGURE 1:**
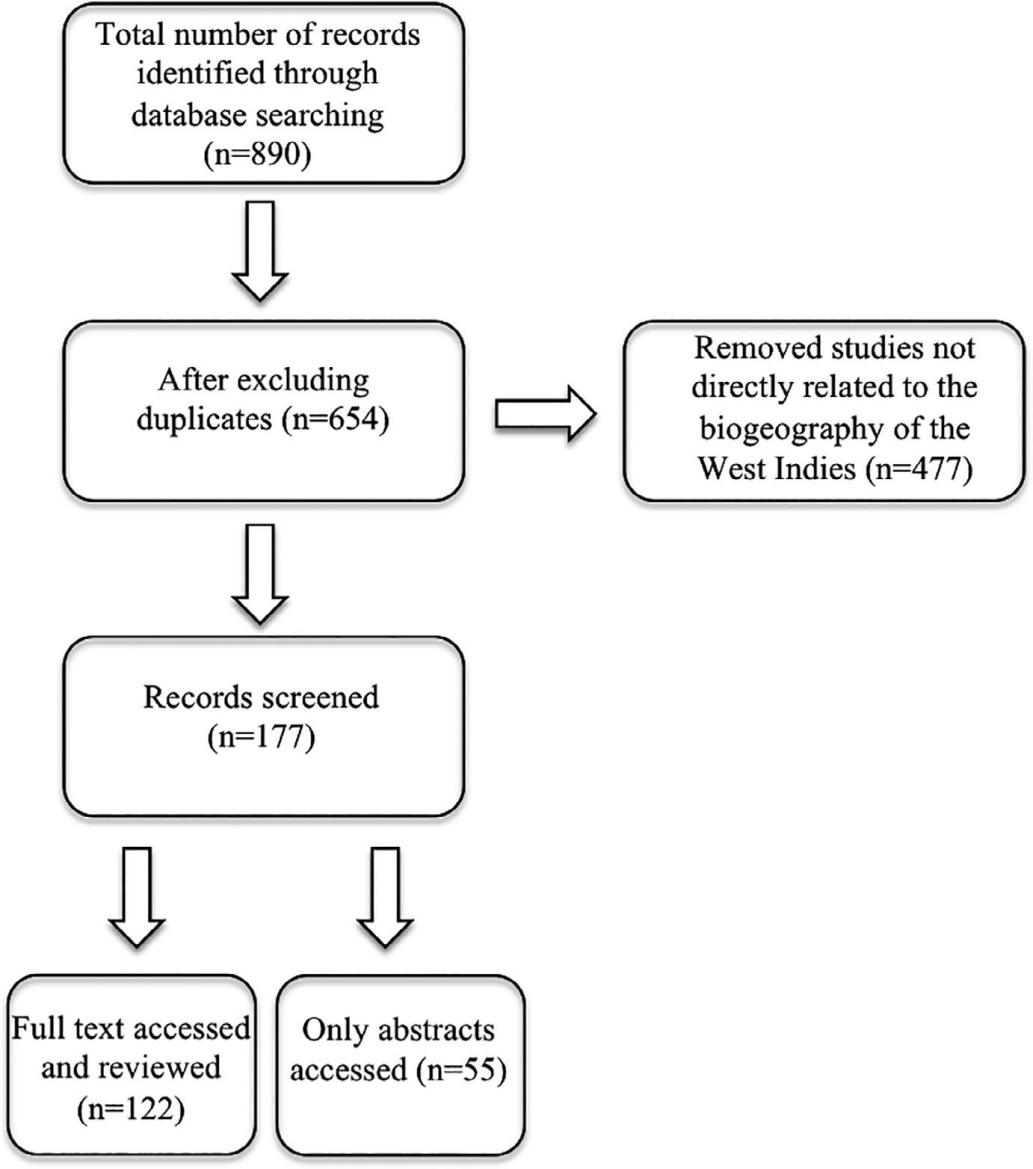
Flow chart of search results and selection process following Dougherty and Shuker (2015). Summary table of studies of the biogeography of the West Indies is available in the Dryad Digital Repository (available at https://doi.org/10.5061/dryad.0k6djh9zv)

In order to provide an objective and reproducible scientific compilation that meets the goals of this review and also guarantees the reproducibility of our results, we conducted a literature search on the Web of Knowledge (Web of Science Core Collection database) through the University of Oklahoma Library website on 17 December 2020. We obtained a total of 890 article records published between 1900 and 2020 using the following key word combinations for the search: “West Indies biogeography” (238 records), “Caribbean biota” (153 records), “Caribbean islands colonization” (234 records), “adaptive radiations Caribbean” (128 records), and “Antilles biodiversity” (131 records). After each search using a specific key word combination, all available outcomes (publications) were assessed and scrutinized based on the topic of the each study (Figure 1).

Here, we only considered publications in which the central theme was related to aspects of the Caribbean biogeography (i.e., historical biogeography, species radiations, phylogenetic, and phylogeographic studies). In addition, we classified the publications according to the specific area coverage for each study, studied group(s) and the analysis of biogeographical trends or biogeographical theories supported by each study (if any).

## 3 GEOLOGY AND GEOLOGICAL HISTORY OF THE WEST INDIES

**FIGURE 2:**
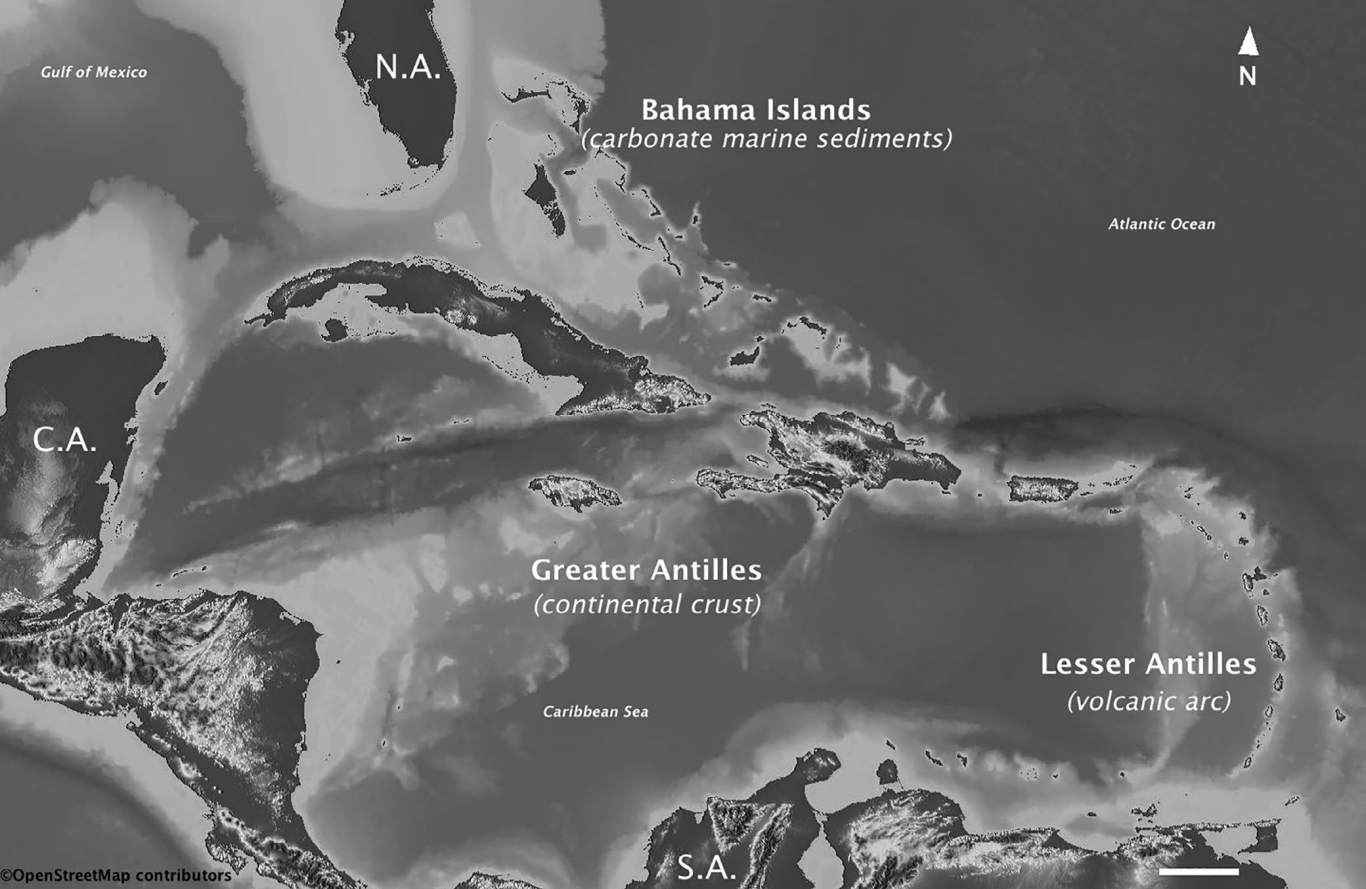
Map of the Caribbean basin showing the three main archipelagoes with corresponding geologic origins that form the West Indies. N.A: North America, C.A: Continental Central America, S.A.: South America. (Scale bar at the bottom represents 200 km)

The three main archipelagoes that form the West Indies (the Bahamas, the Greater Antilles, and the Lesser Antilles) lay in the Caribbean Sea between the continental masses of North and South America (James, 2005). Despite the geographic proximity of these three archipelagoes, the geological origin of their present‐day land territories is totally different from each other and also quite complex (Figure 2). The Bahamas, which are geographically more related to North America, were formed by the accumulation of carbonate marine sediments during lower sea levels in the Pleistocene glacial periods. The origin of the Bahamas platform is completely independent of the Caribbean plate and also unrelated to the origin of the Greater and Lesser Antilles (Meyerhoff & Hatten, 1974). Consequently, the biota of the Bahamas shows relatively low endemism with a flora and fauna mostly derived from North America and the Greater Antilles (Ricklefs & Bermingham, 2008).

Conversely, the Greater Antilles are old islands formed by fragments of continental crust that have been carried to their current position by plate movements (Pindell, 1994). Several parts of the landmasses that currently form Cuba, Hispaniola (Haiti and the Dominican Republic), and Puerto Rico, are thought that have stayed above sea level since the Middle Eocene (Donnelly, 1989; Iturralde‐Vinent & MacPhee, 1999). However, in the formation of the contemporary Greater Antilles periods of extensive inundations caused by sea level changes coupled with movements of the Caribbean plate have resulted in the creation of isolated landmasses that have fragmented and rejoined multiple times resulting in very complex geographical histories (Iturralde‐Vinent & MacPhee, 1999). In fact, Hedges (1996b) stated that the study of the Caribbean biota faces particular challenges due to the difficulty in determining which areas were above or under sea level during the history of the islands.

Although information about the historical relationships among landmasses and geological composition of the islands of the Greater Antilles are yet incomplete, it is broadly established that Hispaniola and Cuba are created by compound regions (Pindell & Dewey, 1982). The geological differences between the western and eastern portions of Cuba represent a clear example of the complex origin of the islands in the Greater Antilles. The eastern part of Cuba, north‐central Hispaniola, and Puerto Rico was probably connected as a single magmatic arc during the Paleocene‐Eocene (Draper & Barros, 1994) and until the Oligocene (Iturralde‐Vinent, 1994). By contrast, the western part of Cuba is exclusive in the Antilles in terms of geological origin (Draper & Barros, 1994) and it is thought to be probably related to the North American Plate (Graham et al., 2000).

The Lesser Antilles are composed of several volcanic islands located at the margin of the Caribbean plate and forming an arc that extends northwest from north of South America to Puerto Rico. This arc of small islands is separated from the Greater Antilles by the Anegada Passage (Santiago‐Valentin & Olmstead, 2004), which is a set of marine basins and deep valleys located across the volcanic arc at the transition between the Greater and the Lesser Antilles (Laurencin et al., 2017). The Lesser Antilles most likely originated about 20 million years ago emerging from above a subduction zone (Wadge, 1994). These small islands which form the Lesser Antilles originated independently from the Greater Antilles, and never had a direct connection with the continent (Ricklefs & Bermingham, 2008). Despite this, they have been basically colonized by the biota from South America (Hedges, 1996b).

Even though the West Indies are geographically located in the neotropics which is relatively stable in terms of temperature, differences if relief among islands lead to some climate heterogeneity that can generate temperature and also precipitation gradients along different elevations (Borhidi, 1996; Rumney & Oliver, 1998). Hispaniola, for instance, contains several mountains with more than 2,000 m in elevation. For example, the highest peak, Pico Duarte in the Dominican Republic, reaches 3,098 m. To some extent, this topographic heterogeneity has led to the evolution of high elevation specialists (mainly amphibians and reptiles) on Hispaniola (Muñoz et al., 2014; Wollenberg et al., 2013). Although less high in elevation, other highland areas reaching over 1,500 m can be also found in other parts of Hispaniola, eastern Cuba, and Jamaica. High elevations have been shown to be an important property in determining the distribution and endemism of island biodiversity (Kallimanis et al., 2010). Such habitat heterogeneity coupled with the relatively large size of islands like Cuba and Hispaniola has been thought to provide niche opportunities for speciation and endemism in several terrestrial groups (Algar & Mahler, 2016; Gentry, 1982; Losos & Schluter, 2000).

## 4 HYPOTHESES THAT EXPLAIN THE ORIGIN OF THE WEST INDIES BIOTA

Despite an intensive debate about models and hypotheses to explain the origin of biodiversity across the Caribbean islands, much of the origin of the biota is still poorly understood and remains an important topics for biogeographers and evolutionary biologists (Čandek et al., 2019; Dávalos, 2004; Reznick et al., 2017; Rodríguez et al., 2010; Santiago‐Valentin & Olmstead, 2004; Tucker et al., 2017; Vázquez‐Miranda et al., 2007). A better understanding of the Caribbean biogeography would also aid investigations of other archipelagoes such as the Philippines, which has an equally complex geological origin (Mitchel et al., 1986). Equally, the multifaceted distinctiveness of the archipelagoes of the West Indies in terms of geology and geological history, varying sizes of the emerged landmasses and their distances to sources of colonists, make them ideal settings to explore the theory of the island biogeography equilibrium and also the influence of in situ speciation processes in the origin of the biodiversity (Ricklefs & Bermingham, 2008).

Three main hypotheses have been historically proposed to explain the origin of biodiversity in the Caribbean islands (Table 1). First, the vicariance model proposed by Rosen (1975), based on terrestrial, freshwater, and marine taxa, suggested that the Proto‐Antilles had a direct connection to mainland South and North America approximately 100–70 million years ago. While this hypothesis has been supported by other authors (Crews & Esposito, 2020; Crother & Guyer, 1996; Page & Lydeard, 1994; Van Ee et al., 2008), the lack of strong geological evidence has led to some controversy around the vicariance model (Hedges, 1996a, 2001; Iturralde‐Vinent, 2006; Williams, 1989). Second, overwater dispersal is another hypothesis that has been proposed to explain the origin of the West Indies biota. This model has received support from several studies that have tested comprehensively many groups of organisms contrasting the fossil records and extant species with precise estimation times of origin of different lineages (Hedges, 2001; Hedges et al., 1992; Williams, 1989). For some terrestrial organisms such as amphibians and reptiles, for instance, transoceanic dispersal likely from South America seems to be the most probable origin of many living groups of these vertebrates in the Caribbean islands (Hedges & Conn, 2012; Heinicke et al., 2007). Both theories, vicariance and overwater dispersal hypotheses, have disagreed with each other with no consensus over many decades. However, some authors have suggested that both processes could have played important roles in the biogeography of different groups in the Caribbean (Hrbek et al., 2007; Newton, 2003).

The third model that explains the biogeography of the Caribbean basin was published by Iturralde‐Vinent and MacPhee (1999). They hypothesized a recent continuous land bridge, GAARlandia (Greater Antilles and Aves Ridge), between north of South America and the Greater Antilles. Such land connection supposedly existed 35–33 million years ago which coincides with intervals of low sea levels that exposed the Aves Ridge allowing the continental South American biota to reach the Caribbean islands. Although there is some support for the GAARlandia hypothesis (Matos‐Maraví et al., 2014; Rican et al., 2013; Weaver et al., 2016), there is still not enough geological evidence to support the land bridge postulate of a continuous dry connection (Ali, 2012). In addition, the absence of many groups of terrestrial mammals in the West Indies do not offer support for a land bridge connection either (Dávalos, 2004; Ricklefs & Bermingham, 2008). Recent molecular evidence has also suggested that GAARlandia did not act as a colonization route for plants from South America to the Antilles (Nieto‐Blazquez et al., 2017).

Certainly, the debate about the colonization of the West Indies biota has moved back and forth between vicariance, land connections, and dispersal in order to explain the origin of the biodiversity in these archipelagoes. In most cases, the momentary establishment of a governing hypothesis has been followed by the publication of influential works that offer at least partial evidence supporting a particular theory. Nevertheless, more intricate biogeographical scenarios that include both dispersal and vicariance models might best explain the formation of the biotas on this region (Heaney, 2007).

## 5 THE WEST INDIES AS SPECIAL SCENARIOS FOR SPECIES RADIATIONS AND ENDEMISM

Islands represent ideal settings to study macroevolutionary processes. Most of the time islands offer novel ecological opportunity for colonists such as abundant food sources and heterogeneous habitats to proliferate (Simpson, 1953). Other advantages that these new environments offer to the initial colonizers are the absence of predators and competitors (Losos & Ricklefs, 2009). Often immigrant lineages radiate filling unoccupied ecological niches in the islands and new species arise through ecological diversification (Schluter, 2000) often in the form of an adaptive radiation (Losos et al., 2006; Schluter, 2000).

Adaptive radiations are extraordinarily important in the origin of biodiversity. Some authors consider this as one of the most important evolutionary processes and likely responsible of a significant part of the ecological and phenotypic diversity of life (Schluter, 2000; Simpson, 1953). There are many classic examples of adaptive radiation that have occurred on islands including Darwin's finches of the Galápagos islands (Grant & Grant, 2002), Hawaiian silverswords (Raven et al., 1992), and Hawaiian honeycreepers (Lovette et al., 2002).

In the West Indies, adaptive radiations have been an important cause of diversification in several groups of organisms. In large and topographically heterogeneous islands like Cuba and Hispaniola, a burst of species formation can be seen in some groups (Ricklefs & Bermingham, 2008). Probably, the most recognized and well‐studied is the case of *Anolis* lizards with more than 150 species in the Caribbean (Glor et al., 2004; Glor et al., 2003; Losos & Schluter, 2000). Anoles have diversified extraordinarily in the Greater Antilles to produce the same set of habitat specialists or ecomorphs in each island (Gavrilets & Losos, 2009; Losos et al., 2006). In the case of the massive adaptive radiation of *Anolis* lizards in the Caribbean, species richness on islands is related to area (Losos, 1996), which is actually a general prediction of the theory of island biogeography (Darlington, 1957; MacArthur & Wilson, 1967) that can also be observed in several other components of the island biota such as plants (Gentry, 1982; Santiago‐Valentin & Olmstead, 2004), spiders (Čandek et al., 2019; Gao & Perry, 2016), butterflies (Matos‐Maraví et al., 2014), and amphibians (Alonso et al., 2012; Rodríguez et al., 2010).

Other groups of extant vertebrates in the Greater Antilles have also experienced high rates of speciation leading to significant endemic biodiversity. Frogs of the genus *Eleutherodactylus* (Eleutherodactylidae), for instance, have been able to radiate in the Greater Antilles making them the dominant group of amphibians in the West Indies (Hedges et al., 2008). In Cuba, for example, 90% (50 species) of the native amphibians are members of this genus (Díaz & Cádiz, 2008; Hedges et al., 2008). The fossil record also offers evidence of multiple radiation events occurred in the past in several lineages of mammals (i.e., primates, sloths, and rodents) of the West Indies (Hedges, 2006). Although extant groups of mammals are not as diverse as the Caribbean herpetofauna, rodents are the richest terrestrial mammal group of the West Indies. Particularly, capromyid rodents (Capromyidae), commonly called hutias, have experienced the largest radiation in the Caribbean islands with eight genera and 32 species that display several ecomorphological adaptations (Fabre et al., 2014). Furthermore, some groups of freshwater fishes also show a significant radiation in the Greater Antilles. Examples of the very interesting patterns of species distribution mainly in the family Poeciliidae are being described later in this review.

Although the majority of the studies on biogeography of the Caribbean islands are primarily focused on animal groups, plant diversity is also outstanding in this region to the point that the Caribbean is considered a distinctive phytogeographic unit within the Neotropics (Gentry, 1982). There are approximately 13,000 seed plants of which about 8,000 are endemic in the West Indies (Acevedo‐Rodríguez & Strong, 2008). The alpha‐diversity of plant species in the Caribbean region is similar to that of Madagascar, and much larger than that of New Caledonia. Over 50% of the vascular plants are endemic to the West Indies, which makes the Caribbean islands a leading hotspot in species‐level endemism (Myers et al., 2000). Particularly in the Greater Antilles, Cuba and Hispaniola, the two largest islands in the Caribbean have the richest flora and highest endemism at the specific and generic level (Gentry, 1982; Santiago‐Valentin & Olmstead, 2004). The smaller islands, Jamaica and Puerto Rico, have less plant diversity, and endemism is mostly at the species level (Santiago‐Valentin & Olmstead, 2004).

Some plant groups show remarkable species radiations in the West Indies such as lineages within the families Melastomataceae (Michelangeli et al., 2008) and Asteraceae (Francisco‐Ortega et al., 2008). Palms (Arecaceae), for example, are also well represented with 135 species of which 121 are endemic (Zona et al., 2007) with most of the endemism concentrated to the Greater Antilles (Roncal et al., 2008). Substrates of serpentine rocks are a very distinctive feature of some islands like Cuba and Puerto Rico (Cedeño‐Maldonado & Breckon, 1996). These environments host an extraordinary biodiversity of unique plants with around 35% of all endemic genera known in Cuba confined to serpentine (Berazaín‐Iturralde, 1976; Brooks, 1987).

Unfortunately, there is an unbalanced number of biogeographical studies creating taxonomic bias in the study of the West Indian biota. After reviewing the available literature in the Web of Science Core Collection database using different key word combinations, we found that most of the studies that examine the biogeography of the West Indies and the Caribbean islands, in general, involve terrestrial groups. Of the 890 publications in the field that resulted from our initial search and after a critical appraisal, we identified 177 to be relevant to the study of the biogeography of the West Indies. The scientific work in this field has been growing notably. For example, during the last 5 years alone 62 studies have been published, representing 35% of all the literature compiled in our review. Most of the publications we found (162 studies, 91.5%) were related in the study of terrestrial organisms, only a small portion covered aquatic groups (13 studies, 7.3%) and only a couple review contributions (two studies, 1.2%) covered the study of both terrestrial and aquatic organisms. We identified vertebrates (mainly terrestrial vertebrates, which included birds and bats) as the most studied group (102 studies, 57.6%), followed by invertebrates (46 studies, 23.7%) and plants (28 studies, 15.8%). In studies that tested biogeographical models of the origin of the West Indian biota, we found that several of them included more than one single model to explain the biodiversity found on the islands. However, overwater dispersal emerged as the most relevant mechanism of colonization of the West Indies not only for organisms with good dispersal abilities (flying insects, birds, bats, etc.), but also for species with poor dispersal abilities, mainly through rafting flotsam moved by large storms and marine currents.

Our results show that studies on groups such as plants, terrestrial or aquatic arthropods, and freshwater fishes are vastly underrepresented relative to their taxonomic diversity. One of the most important steps to fill the information gap on these groups (mainly in aquatic organisms) requires the preparation of specialists with the necessary knowledge and skills to document biodiversity in these scarcely studied groups. It is also imperative to perform integrative analyses that uncover the possible origins of these groups in the Caribbean islands and also evolutionary mechanisms leading to speciation.

## 6 SPECIES RADIATION IN AQUATIC ENVIRONMENTS OF THE WEST INDIES: THE CASE OF LIVEBEARING FISHES IN THE GREATER ANTILLES

Certainly, the classic example of adaptive radiation in aquatic vertebrates is the case of cichlid fishes (Cichlidae) from the East African Great Lakes (Turner et al., 2001). The explosive speciation rate in this group is known to be responsible for generating an outstanding diversity in behavior, coloration, body shapes, and a huge diversity of trophic and other ecological specializations (Fryer & Iles, 1972; Kornfield & Smith, 2000; Malinsky et al., 2018; Meier et al., 2017; Meyer, 1993; Seehausen, 2015; Turner, 2007).

Inland water bodies in the West Indies are relatively small even on the larger islands (Vergara, 1992). This size limitation imposes some constraints for species diversification in aquatic groups. However, although not quite as rampant as the case of the cichlid radiation in the African rift valley lakes, the West Indies exhibit some examples of young adaptive radiations in fishes. For instance, three species of *Cyprinodon* (Cyprinodontidae) that coexist sympatrically in hypersaline lagoons within the San Salvador Island, Bahamas have been described to have trophic partitioning (Martin, 2016; Martin & Wainwright, 2013). *C. variegatus*, a species with broad geographic distribution and with detritivorous feeding habits, is thought to have given origin to this small Bahamian radiation (Hernandez et al., 2018). The other two species are diet specialists: *C. brontotheroides* specializes in consuming hard prey (i.e., ostracods, gasteropods) and *C. desquamator* a specialized scale‐eater (Martin & Wainwright, 2013).

Nonetheless, livebearing fishes (Poeciliidae) is the group of freshwater vertebrates that has experience the highest radiation in aquatic environments of the West Indies with three endemic genera (*Girardinus*, *Quintana*, and *Limia*) distributed in the Antilles (Doadrio et al., 2009; Hamilton, 2001; Reznick et al., 2017; Rosen & Bailey, 1963). The genus *Gambusia* is also represented in the archipelagoes of the Greater Antilles and the Bahamas where ecological speciation has been shown to be associated with divergent predation regimes (Langerhans et al., 2007).

Poeciliids represent an ideal model to study the origin of the Caribbean biota (García‐Machado et al., 2020). They are the dominant group of freshwater fishes in the insular Caribbean and also exhibit an extraordinary diversity in continental Middle America (Rosen & Bailey, 1963; Vergara, 1992). Furthermore, this group of fishes offers a complex scenario when analyzing colonization events in the Caribbean as compared to terrestrial or other freshwater groups. Several poeciliid species show varying levels of tolerance to water salinity, which could made possible overseas dispersal from the mainland (Briggs, 1984; Darlington, 1938; Myers, 1938; Rosen & Bailey, 1963). In fact, much of the controversy between dispersal and vicariance advocates in relation to the origin of the Antillean freshwater fish fauna revolves around the fact that poeciliids are a group that ecologically lies between terrestrial and purely freshwater species so that both models can explain the biogeographic distribution observed in this group (Hrbek et al., 2007; Palacios et al., 2016; Reznick et al., 2017; Rivas, 1958; Rosen & Bailey, 1963; Weaver et al., 2016).

**FIGURE 3:**
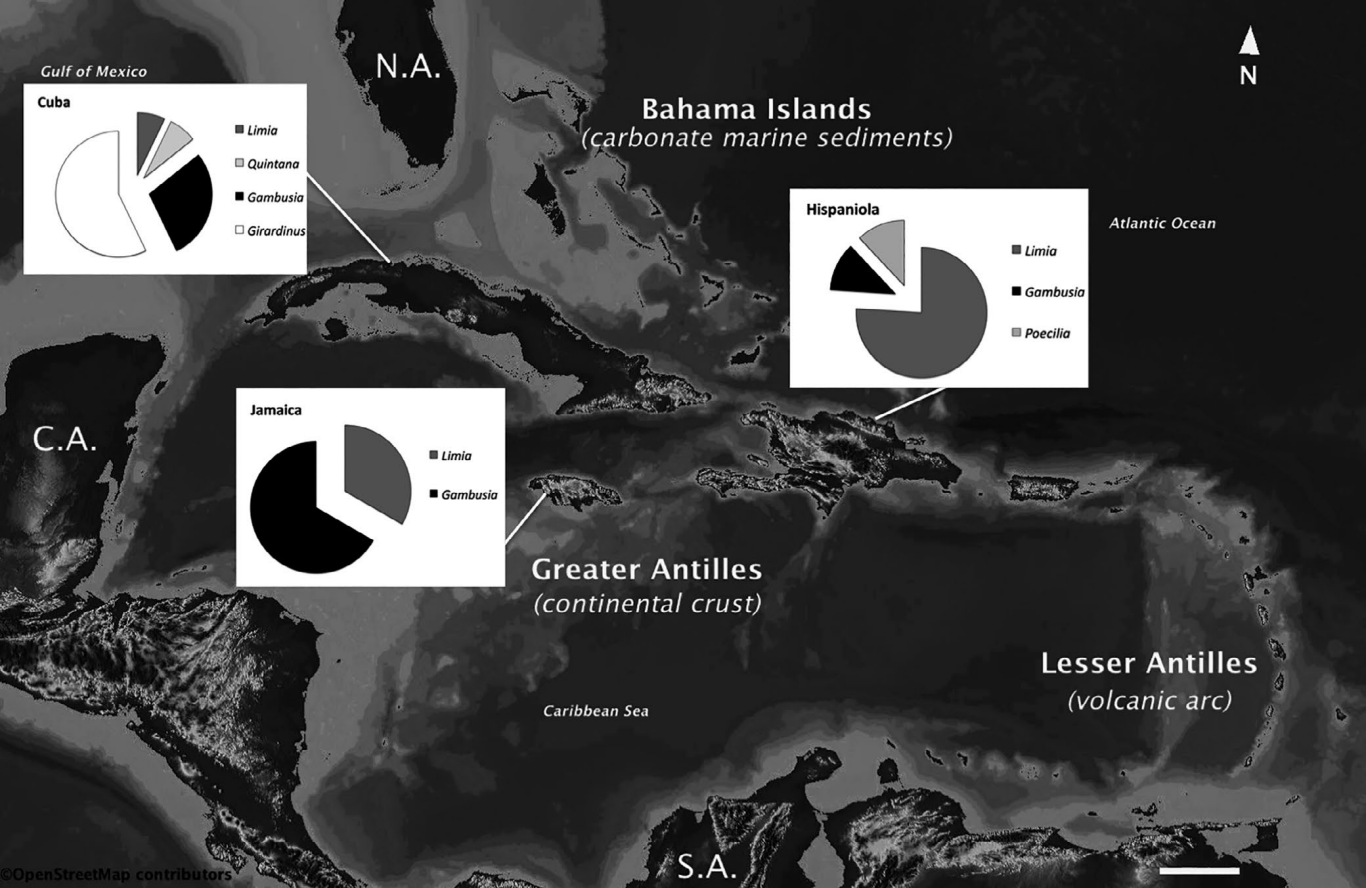
Map of the West Indies showing the number of endemic species by genera in the Greater Antilles. Hispaniola shows the largest radiation since a single lineage (*Limia*) has radiated in 19 known species. Puerto Rico has a very limited freshwater fauna with no endemic species of poeciliids. (Scale bar at the bottom represents 200 km)

Even though the general prediction from theory that relates species richness to island size is valid for livebearing fishes in the West Indies, where larger islands have higher number of species mainly due to in situ speciation (Furness et al., 2016), the origin of the different the lineages and the species composition among genera show a very interesting pattern in the Greater Antilles (Figure 3).

The oldest radiation event documented for poeciliids in the West Indies was related to the ancestors of today's genus *Girardinus* and its sister genus *Quintana*, both endemic to Cuba (Doadrio et al., 2009; Hrbek et al., 2007; Reznick et al., 2017; Rivas, 1958). *Girardinus* shows a higher diversity in western Cuba and it has eight described species (Lucinda, 2003): *G. metallicus* Poey, 1854; *G. uninotatus* Poey, 1860; *G. creolus* Garman, 1895; *G. denticulatus* Garman, 1895; *G. cubensis* (Eigenmann, 1903); *G. falcatus* (Eigenmann, 1903); *G. microdactylus* Rivas, 1944 and *G. rivasi* Barus and Wohlgemuth, 1994. *Quintana* is a monotypic genus represented by the species *Quintana atrizona* Hubbs, 1834, which is only distributed in southwestern Cuba including Isla de la Juventud. Rivas (1958) proposed that the ancestor of *Girardinus* colonized Cuba from Yucatan during upper Miocene‐Pliocene via a land bridge that connected western Cuba to the Yucatan peninsula. Rosen and Bailey (1963) also supported a Yucatan‐Cuba land connection and considered *Quintana* and the genus *Carlhubbsia* from eastern Yucatan sister genera of *Girardinus*. Recent studies have questioned the Yucatan‐Cuba land connection and other alternative models (i.e., GAARlandia model) have been proposed to explain the origin this lineage and poeciliids, in general, in the Greater Antilles. Molecular evidences suggest that common ancestors of these taxa dispersed from South America approximately 45–40 million years ago (Hrbek et al., 2007; Reznick et al., 2017).

Another dispersal event that is relevant to the origin of the Caribbean ichthyofauna occurred also from South America about 22–16 million years ago and originated the radiation of the genus *Limia* (genus *Poecilia* subgenus *Limia* sensu Reznick et al. (2017)) (Palacios et al., 2016; Reznick et al., 2017; Weaver et al., 2016). Palacios et al. (2016) also showed that three endemic *Poecilia* species to Hispaniola: *P. dominicensis*, *P. elegans*, and *P. hispaniolae* were closely related and basal to the *Limia* clade which might suggest that this triad of species is the result of a second colonization event from South America (Reznick et al., 2017). Certainly, the speciation process in the genus *Limia* has resulted in the largest radiation of the family Poeciliidae in the West Indies. So far 22 species of this genus have been described from Cuba, Hispaniola, Jamaica, and Grand Cayman. The center of radiation of *Limia* is located on Hispaniola with 19 species while only one endemic species from Cuba, Jamaica, and Grand Cayman each are currently known (Burgess & Franz, 1989; Hamilton, 2001; Rodriguez‐Silva et al., 2020; Rodriguez‐Silva & Weaver, 2020; Weaver et al., 2016). This divergent species composition, mainly in the cases of Cuba and Hispaniola, is not predicted by the theory of island biogeography as the number of available ecological niches and island size is likely the same in both islands. A brief analysis of probable causes of the deviation from the species‐area relationship in this genus and in livebearing fishes, in general, is provided in the next section of this paper.

Finally, the most recent dispersal event that is known for poeciliids of the West Indies occurred between 1 and 11 million years ago and involved the genus *Gambusia* (Reznick et al., 2017). Both classic revisions of the genus (Rauchenberger, 1989; Rivas, 1963) and recent studies suggest that the ancestor of *Gambusia* arrived in the Caribbean islands from Central America likely via overwater dispersal (Hrbek et al., 2007; Lydeard et al., 1995; Palacios et al., 2016; Reznick et al., 2017).

The debate over the complex origin of the livebearing fishes in the West Indies as well as its implication in the disjunctive distribution patterns observed in the area has been broadly discussed for a long time (Darlington, 1938; Myers, 1938; Rivas, 1958; Rosen & Bailey, 1963). Yet today, several questions still exist on how members of the family Poeciliidae have radiated, colonizing almost every available fresh and brackish water environment in the Caribbean islands.

## 7 SPECIES‐AREA RELATIONSHIP (SAR): THE TRENDS OF TERRESTRIAL VERSUS AQUATIC GROUPS IN THE ANTILLES

The trend of larger islands to containing more species and smaller islands containing fewer species emerges as a rule in general terms in the Caribbean islands (Darlington, 1957; Losos, 1996; Losos & Schluter, 2000; Ricklefs & Bermingham, 2004), and this has been a core principle of the classical island biogeography theory in general (MacArthur & Wilson, 1967). While a one‐dimensional interpretation of the SAR essentially focused on the correlation between island area and species richness has been widely adopted, it has been shown that ignoring functional differences among species may be a simplistic approach as it considers all species ecologically similar (Hubbell, 2001). Thus, the niche theory, which focuses mostly in the importance environmental heterogeneity and niche partitioning as key drivers of species richness, has emerged as another major hypothesis for our understanding of the SAR (Hortal et al., 2009). Since elements of the two theories likely act together, an integrated view of both has been lately proposed to explain diversity patterns observed on islands (Franzén et al., 2012; Kadmon & Allouche, 2007; Lomolino & Brown, 2009).

To this day, integrative approaches addressing the influence of the two SAR components on species richness in the Antilles are limited, and most of them have been concerning terrestrial organisms (Losos, 1996, 2009; Rodríguez‐Durán & Kunz, 2001; Weerd et al., 2016). On the other hand, studies of aquatic groups (mainly freshwater species) are very rare in the literature. These two factors, the scarce number of studies that offer an integrated perspective of the SAR in the insular Caribbean, as well as the unbalanced taxonomic treatment for the analysis of terrestrial groups over aquatic species, has led to generalizations about the SAR without deeper examinations of how island area together with environmental heterogeneity and niche partitioning may affect species richness in terrestrial (excluding some studies in Caribbean anoles e.g., Losos (2009)) and aquatic environments differently.

**FIGURE 4:**
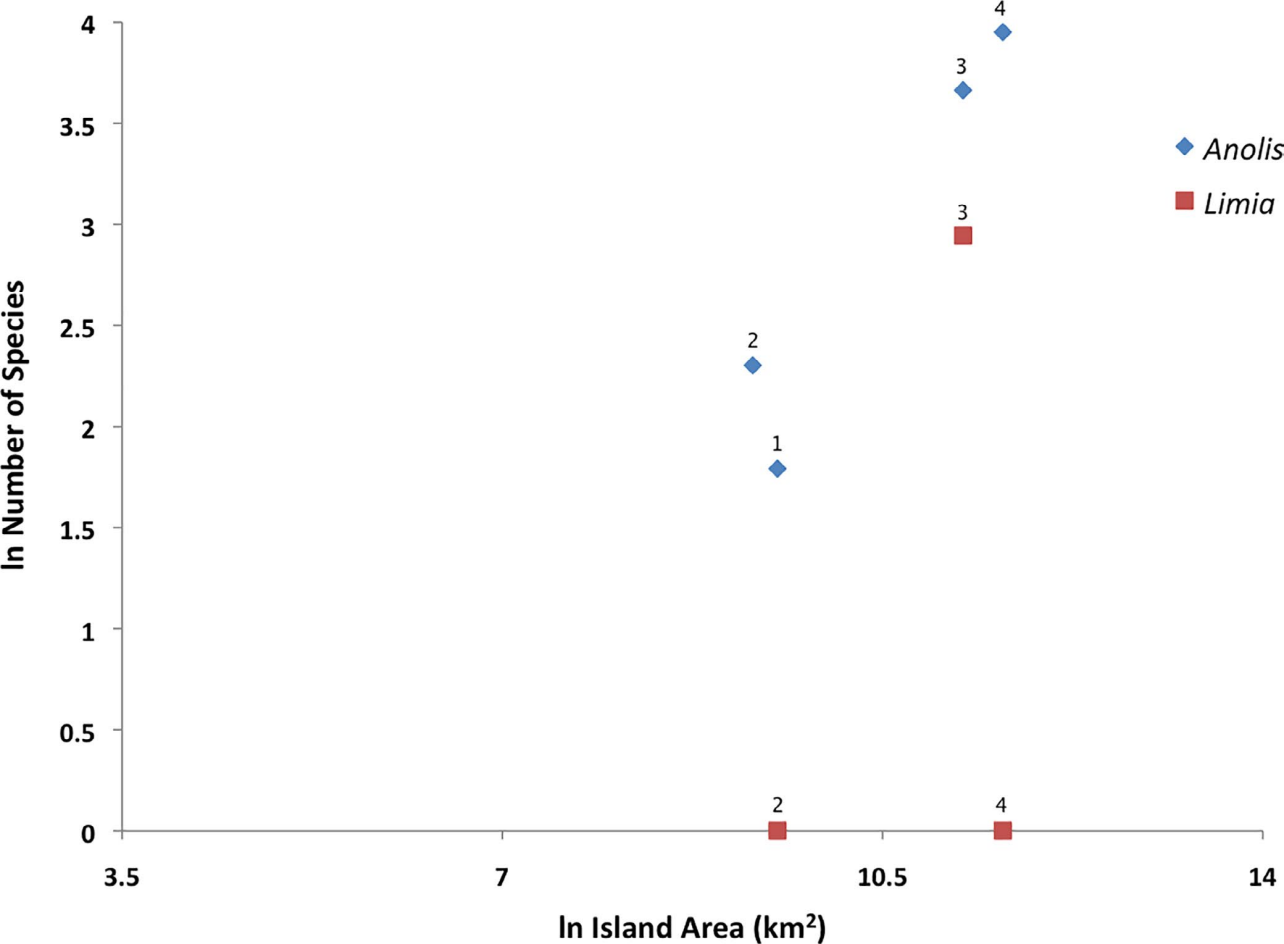
Diversification of *Anolis* lizards and livebearing fishes of the genus *Limia* in the Greater Antilles. The ln of the number of species of *Anolis* is strongly correlated with ln island area (*r* = 0.96), but not the ln of the number of species of *Limia* with ln island area (*r* = 0.35). 1: Puerto Rico, 2: Jamaica, 3: Hispaniola, 4: Cuba. Data from Algar and Mahler (2016) and Furness et al. (2016)

Probably, the most contrasting example about diverging speciation patterns relative to island size in aquatic versus terrestrial organisms in the Greater Antilles is the radiation process of livebearing fishes of the genus *Limia*. It has been very different from what is currently observed in anole lizards (Figure 4) in which larger islands display similar species richness (Hedges, 1996a; Losos, 2009). Hence, it is intriguing to ask which mechanisms have been influencing the speciation of *Limia* in such extreme different ways and what could explain this lopsided distribution.

Other contrasting trends between island size and species richness in terrestrial versus aquatic habitats have been observed in freshwater macroinvertebrates of the Lesser Antilles. In a study of the biodiversity of freshwater macroinvertebrates in 14 small Caribbean islands, Bass (2003) found that the patterns of species richness showed some divergence with those observed in the case of the Caribbean herpetofauna (Darlington, 1957; Hedges, 1996a; Losos, 2009). According to the results of his study, Bass (2003) proposed that number and height of elevations in each island might also be considered an important factor influencing the number of macroinvertebrate species present because of the direct, positive relationship between elevation, rainfall, and number of freshwater habitats. These findings show that the classical island biogeography theory alone may not completely explain the observed species richness patterns and also suggest that ecological opportunity could outweigh the importance of island size in speciation.

In our study, we ran a similar comparison to determine the relationship between island size, the presence of elevations, and species richness of livebearing fishes in the Greater Antilles. Our analysis reveals a similar pattern observed in aquatic invertebrates of the Lesser Antilles (Table 2) showing that not only the island size but also mountainous reliefs may be an important factors that determine the number of livebearing fishes species in the Greater Antilles.

**TABLE 2:** Livebearing fish species richness in the Greater Antilles in relation to island size, maximum elevation, and average elevation

Island	Number of species	Area (km^2^)	Maximum elevation (m)	Area × maximum elevation	Average elevation (m)	Area × average elevation
Cuba	16	109,884	1,974	216,911,016	137	15,054,108
Hispaniola	24	76,192	3,175	241,909,600	424	35,305,408
Jamaica	4	11,420	2,256	25,763,520	340	3,882,800
Puerto Rico	0	9,104	1,338	12,181,152	261	2,376,144

A Spearman correlation showed a positive, yet nonsignificant relationship between island size and the number of livebearing fish species (*r*_s_ = 0.80, *n* = 4, *p* = 0.20). However, when accounting for the effect of island area combined with elevation there was a positive, significant relationship for both island size × maximum elevation and island size × average elevation and the number of livebearing fish species in the Greater Antilles (*r*_s_ = 1, *n* = 4, *p* < 0.001).

## 8 HUMAN IMPACT AND THREATS TO THE BIODIVERSITY OF THE WEST INDIES

The growth rate of the human population has exponentially increased worldwide becoming an evolutionary force of extraordinary pressure on global biodiversity (Palumbi, 2001). The level of stress and exploitation that human activities exert on natural ecosystems is so high that they can cause evolutionary changes sometimes within a few hundred years (Hendry & Kinnison, 1999; Reznick & Ghalambor, 2001). Factors such as global climate change (Omann et al., 2009) and its direct effect on sea‐level rise (Courchamp et al., 2014), habitat degradation and fragmentation (Brooks et al., 2002; Hansky, 2011), and exotic species (Groom et al., 2005; Sala et al., 1999; Tye et al., 2018) have been shown to lead to new selection pressures on biodiversity causing an increase in the risk of extinction of several groups. Although all these threats affect the biodiversity worldwide, there some intrinsic features of islands make them more fragile and vulnerable than the continental biota (Loope et al., 1988; Paulay, 1994; Vermeij, 1991; Vitousek, 1988). According to MacArthur and Wilson (1967), natural dynamics of colonization and extinction have occurred for million years on islands where an equilibrium is established once rates of colonization and extinction are equal. In the West Indies, the existing biodiversity that has evolved surviving major, sometimes catastrophic, geological events is now facing an extinction rate without precedent due to anthropogenic activities mainly after the human occupation of the West Indies (Ricklefs & Bermingham, 2008).

For example, the percent annual rates of deforestation in the Caribbean are the highest among all biodiversity hotspots in the world, which threatens most endemic plant and animal species (Food & Agriculture Organization, 1997). Haiti, for instance, is one of the most deforested countries in the world with less than 1% of its original primary forest (Hedges et al., 2018). Certainly, the effect of habitat destruction has been a key factor in the extinction of Caribbean terrestrial vertebrates and particularly endemic and specialized mammals (i.e., cave‐dwelling bats species) that are either threatened or already extinct (Brooks et al., 2002; Morgan, 2001; Woods, 1989). Habitat loss has been also reported as one of the major causes of species decline and extinction in amphibians (Hedges, 1993; Hedges et al., 2018), reptiles (Hedges et al., 2018), and birds (Devenish‐Nelson et al., 2019).

Human‐mediated species introduction, intentionally or accidentally, is also the foremost cause of biodiversity loss in the West Indian archipelagoes (Ricklefs & Bermingham, 2008). Frequently, these exotic species share ecological niches with the native ones causing negative effects on the biodiversity. Although not always well documented in all groups, the impacts of introduced species include predation of endemic species, disease transmission, and competition for resources (Courchamp et al., 2003; Kelly et al., 2009; Rasambainarivo & Goodman, 2019). Even though introduced species represent a threat to the biodiversity worldwide, recent studies have shown that the magnitude of the contemporary effects of species translocations on island biogeography is determined by the economic isolation of human populations (Furness et al., 2016; Helmus et al., 2014).

There are countless examples of introduced species in the archipelagoes of the West Indies. Nevertheless, empirical approaches that assess distribution ranges and the damaging effects of exotic species are not very abundant. One of these is the case of the mongoose (*Herpestes javanicus*), which is widespread in most Caribbean islands and is known to drastically affect native species of birds (Horst et al., 2001). Another example is the introduction of the common green iguana (*Iguana iguana*) in the Lesser Antilles that has displaced other native iguana species through competition and hybridization (Vuillaume et al., 2015). Freshwater environments in the West Indies are also under pressure of introduced species as several alien fishes are being introduced as source of human protein (Ponce de León et al., 2013; Rodríguez‐Barreras & Zapata‐Arroyo, 2019), as biological control of mosquito larvae (Deacon et al., 2011), and also as result of the pet trade (Bunkley‐Williams et al., 1994; Rodríguez‐Barreras et al., 2020). A coordinated conservation effort in the Caribbean region is a challenging task because of the lack of financial resources and the presence of many islands administrated by independent governments with their own conservation strategies and priorities (Ricklefs & Bermingham, 2008), as well as language barriers. However, the conservation of the biota in the West Indies requires immediate action in view of the increasing threats to biodiversity. Collaborative work that involves both local and foreign researchers will definitely help in the design of urgent management plans to ameliorate the negative effects of human actions on native species and ecosystems.

Definitely, studies of biogeographic patterns of the Caribbean biota have been one of the most fertile topics in investigations of island biogeography. However, still limited, inconclusive information exists regarding the origins and determinants of posterior diversification for many groups in the West Indies. The characteristic archipelagoes that are found in this geographic region offer countless possibilities to develop empirical microevoloutionary studies to understand the origin and posterior diversification of the insular Caribbean biota.

## CONFLICT OF INTEREST

The authors have declared that no competing interests exist.

## AUTHOR CONTRIBUTIONS

**Rodet Rodriguez‐Silva:** Conceptualization (equal); data curation (lead); formal analysis (equal); funding acquisition (equal); investigation (equal); methodology (equal); writing – original draft (lead); writing – review and editing (equal). **Ingo Schlupp:** Conceptualization (equal); formal analysis (equal); funding acquisition (equal); investigation (equal); methodology (equal); writing – review and editing (equal).

## Data Availability

Data curated from the Web of Knowledge (Web of Science Core Collection database) that was analyzed in this study is available in the Dryad Digital Repository (available at https://doi.org/10.5061/dryad.0k6djh9zv).
